# A complete genome sequence of *Pseudomonas amygdali* pathovar *lachrymans* YM7902

**DOI:** 10.1128/mra.00682-24

**Published:** 2024-10-22

**Authors:** Audrey Sweten, David Baltrus

**Affiliations:** 1 School of Animal and Comparative Biomedical Sciences, University of Arizona, Tucson, Arizona, USA; 2 School of Plant Sciences, University of Arizona, Tucson, Arizona, USA; University of Southern California, Los Angeles, California, USA

**Keywords:** *Pseudomonas syringae*, *Cucumis sativus*, phytopathogen

## Abstract

*Pseudomonas amygdali* pathovar *lachrymans* YM7902 was originally isolated as a pathogen of cucumber in Japan. Here, we report a nearly complete genome sequence for this strain, assembled using a hybrid approach combining Illumina paired-end reads and longer reads sequenced using technology from Oxford Nanopore.

## ANNOUNCEMENT

*Pseudomonas syringae* (*sensu lato*) is a species complex containing numerous lineages that can cause a variety of diseases on plants in both agricultural and natural systems (1). Although numerous *Pseudomonas syringae* genome assemblies have already been reported, sequencing of additional isolates can inform our understanding of evolutionary dynamics and diversity of virulence pathways for this phytopathogen (2). *Pseudomonas amygdali* pv. *lachrymans* strain YM7902 was originally isolated from diseased cucumbers (*Cucumis sativus*) in 1979 in Japan, and the isolate described herein was obtained from the Ministry of Agriculture, Forestry, and Fisheries (MAFF) in Japan, where it is listed under accession MAFF 730057 (2). Once obtained, a lyophilized stock was resuspended in King’s Media B (KB) and plated onto KB agar plates without antibiotics. From this original plating, a single colony was isolated and picked to a 2 mL culture of KB media, grown overnight at 27°C in a shaking incubator, and frozen as a stock culture the next day. We streaked a subsample of this frozen stock to KB agar plates to obtain a single colony isolate, which was then picked to 2 mL of KB media and grown overnight as described above, after which the genomic DNA was isolated using a Wizard DNA Extraction Kit (Promega, Madison, WI, USA) following the manufacturer’s directions without size selection or RNase treatment. The same DNA isolation was used for both Illumina and Nanopore sequencing.

DNA was sequenced by SeqCenter (Pittsburgh, PA, USA) following their standard workflow for library preparation and read trimming. For Illumina sequencing, the Illumina DNA Prep Kit and IDT 10 bp UDI indices were used to prepare a sample library of 150 bp paired-end reads, which was then sequenced on an Illumina NextSeq 2000 producing a total of 2,843,974 reads totaling 416,590,372 bp at 62× depth. For Nanopore sequencing, libraries were prepared using a Ligation Sequencing Kit with the NEBNext Companion Module in addition to Native Barcode Kits according to the manufacturer’s specifications. All samples were run on Nanopore R9.4.1 flow cells, and MinION Mk1B device and the post-sequencing program Guppy (version 5.0.16) were used for high accuracy basecalling and demultiplexing. Nanopore sequencing generated 59,716 reads totaling 309,715,335 bp at 45× coverage. The *N*50 for the nanopore reads was 13,512 bp. DNA was neither sheared nor size-selected for Nanopore sequencing.

A hybrid assembly was generated by combining both read types in Unicycler version 0.4.8 (3). Refer to Table 1 for the replicon characteristics of strain YM7902 arising from this assembly. The total DNA length of the YM7902 hybrid assembly was 6,759,796 bp. The chromosome and five out of six plasmids within YM7902 were determined to be circular and were rotated by the Unicycler pipeline. The chromosome for YM7902 was annotated by the PGAP at GenBank pipeline with 5,894 predicted unique genes (4). Default parameters were used for all software.

**TABLE 1:** Sequencing and assembly data for *Pseudomonas amygdali* YM7902 strain.

Replicon	Accession	Size	GC content (%)	Coverage (×)	Circular by Unicycler?
Chromosome	CP127045.1	6,122,931	58	1.00	Yes
pPlaYM7902A	CP127046.1	445,381	53	1.18	Yes
pPlaYM7902B	CP127047.1	77,752	55.5	5.38	Yes
pPlaYM7902C	CP127048.1	53,818	55	N/A^*a*^	No
pPlaYM7902D	CP127049.1	47,967	54	8.49	Yes
pPlaYM7902E	CP127050.1	10,027	53.5	13.23	Yes
pPlaYM7902F	CP127051.1	1,906	57	793.57	Yes

^*a*^ N/A represents a lack of rough estimated copy number because the replicon was not circularized.

## Data Availability

The genome for strain YM7902 is indexed at GenBank and is associated with BioProject accession number PRJNA976399. The chromosomal sequence for strain YM7902 can be found under GenBank accession number GCA_030252675.1. The Illumina reads can be found under SRA accession number SRX20517712. The Oxford Nanopore can be found under SRA accession number SRX20517713.
